# Green Synthesis of Homogeneous Gold Nanoparticles Using *Sargassum* spp. Extracts and Their Enhanced Catalytic Activity for Organic Dyes

**DOI:** 10.3390/toxics9110280

**Published:** 2021-10-28

**Authors:** J. Luis López-Miranda, Gustavo A. Molina, Rodrigo Esparza, Marlen Alexis González-Reyna, Rodolfo Silva, Miriam Estévez

**Affiliations:** 1 Centro de Física Aplicada y Tecnología Avanzada, Universidad Nacional Autónoma de México, Boulevard Juriquilla 3001, Querétaro 76230, Mexico; lopezfim@fata.unam.mx (J.L.L.-M.); gustavomolina21@gmail.com (G.A.M.); resparza@fata.unam.mx (R.E.); marlengonzalez@fata.unam.mx (M.A.G.-R.); 2 Instituto de Ingeniería, Universidad Nacional Autónoma de México, Edificio 17, Ciudad Universitaria, Coyoacán, Mexico City 04510, Mexico; rsilvac@iingen.unam.mx

**Keywords:** gold nanoparticles, green synthesis, *Sargassum* spp., organic dye, water treatment

## Abstract

*Sargassum* species-based extracts were used to carry out the synthesis of homogeneous gold nanoparticles. Various techniques were used to determine the characteristics and composition of the nanoparticles. The UV-Vis results showed that the 50% water/ethanol extract had the most reducing agents and stabilizers. Therefore, this type of extract was used to synthesize nanoparticles and for their subsequent characterization. Crystallinity and crystal size were evaluated using X-ray diffraction. Size and morphology were analyzed using scanning electron microscopy, showing that the gold nanoparticles were mostly spherical, with a size range of 15–30 nm. The catalytic activity of the gold nanoparticles was evaluated through the degradation of organic dyes: methylene blue, methyl orange, and methyl red. The degradation rates were different, depending on the nature of each dye, the simplest to degrade was methylene blue and methyl red was the most difficult to degrade. The results indicated that the use of *Sargassum* spp. for the synthesis of gold nanoparticles has potential in the remediation of water that is contaminated with organic dyes. Moreover, given the recent serious environmental and economic problems caused by the overpopulation of *Sargassum* spp. in the Mexican Caribbean, the findings hold promise for their practical and sustainable use in the synthesis of nanomaterials.

## 1. Introduction

The pollution of water by organic dyes is a serious problem worldwide. These substances are widely used by the paper, textile, pharmaceutical, and cosmetic industries [1,2] to provide color to a given material or object. However, the effluents from these industries pass into rivers and lakes, and cause contamination of ecosystems, affecting many living beings [3]. The removal of organic dyes is of great interest because they are highly toxic, carcinogenic, and mutagenic compounds [4,5]. They are highly soluble in water and have great stability, making removal difficult [6]. Various methods exist for their removal, including absorption, filtration, osmosis, and flocculation [7], but these are expensive, complex, and inefficient.

The degradation of these substances using nanoparticles has been reported as an efficient and relatively simple process [8,9]. Silver, gold, copper, and platinum nanoparticles have been shown to degrade organic dyes [6,10]. Gold nanoparticles (AuNPs) have been evaluated for their excellent optical, chemical, and catalytic properties [11,12,13]. Many of these properties are attributed to their high surface-to-volume ratio. In addition, their compatibility and low toxicity make AuNPs a material with a wide range of applications in catalysis, sensing, and biomedicine [14]. AuNPs can be obtained by several chemical, physical, and biological methods [15,16]. The biological approach has attracted special interest when AuNPs are intended for use in areas such as biomedicine and the environment [17,18,19], as these methods are safe, non-toxic, and eco-friendly [20,21]. In biological synthesis methods, also called biosynthesis or green synthesis, an organism is used to reduce metal ions and subsequent stabilization of the nanoparticles [22,23]. Bacteria, fungi, plants, and algae have been reported for these purposes [10,24,25]. The simplicity and low cost of using algae makes it an attractive option.

In this work, the *Sargassum* spp. that exist in the Mexican Caribbean were used: *Fluitans* and *Natans* [26]. These species have a high content of antioxidant compounds that can synthesize nanoparticles [26,27]. In addition, since large quantities of *Sargassum* spp. have recently been beached on the Mexican Caribbean, this work has the further attraction of, hopefully, alleviating some of the serious environmental and economic problems this causes [28,29,30]. The *Sargassum* spp. found on the sea surface obstruct the passage of light, leading to alterations in the photosynthesis cycles of marine flora and fauna [28]. Economic consequences of the influx include the significant reduction in tourism in the area and the costs of collecting and processing these algae [28,31]. Thus, the characteristics, properties, and compounds of *Sargassum* spp. are being exploited in various useful applications to reduce its overpopulation [32,33].

This work examines the synthesis of AuNPs using extracts prepared with *Sargassum* spp. from the Mexican Caribbean. The catalytic properties of the AuNPs obtained were evaluated through the degradation of organic dyes dissolved in water.

## 2. Materials and Methods

### 2.1. Materials

The *Sargassum* spp. used in this study was collected on the Mexican Caribbean coast in June 2021 and cleaned on-site of epiphytes and sand. Then, at the laboratory, the Sargassum was thoroughly washed with tap water and distilled water to remove litter and impurities, then dried in the sun for 48 h to remove all moisture. After all the Sargassum was dried, it was pulverized using a 1000 watts domestic blender and washed again with distilled water. The water was then removed using Whatman #41 filter paper, and the Sargassum was dried using an air oven at 40 °C. Finally, the powder was homogenized using a metallic sieve of 150 µm and stored at room temperature in a dark dry place in sealed plastic bags until it was used.

The precursor salt used was analytical grade HAuCl_4,_ purchased from Sigma. The methylene blue, methyl orange, and methyl red used to evaluate the catalytic properties and the NaBH_4_ reagent, employed for catalytic evaluation, were also purchased from Sigma. Finally, the different aqueous solutions and dilutions were prepared using distilled water.

### 2.2. Extract Preparation

The extracts were prepared by mixing 1 g of *Sargassum* spp. with 50 mL of solvent (water, ethanol, or 50% water/ethanol) using magnetic stirring at 60 °C for 30 min. Subsequently, the liquid phase was recovered by filtration using Whatman #41 filter paper.

### 2.3. Synthesis of AuNPs

The AuNPs synthesis was carried out by mixing the *Sargassum* spp. extract with an aqueous solution of HAuCl_4_ (5 mM). All the experiments were carried out at room temperature and using magnetic stirring. The reduction capacity of *Sargassum* spp. extracts obtained with different solvents were evaluated. In this case, 4 mL of HAuCl_4_ aqueous solution were mixed with 2 mL of each extract. According to UV-Vis results, the most efficient sample was selected for the experiments on the variation of extract/salt volumetric ratio (1:3, 1:2, 1:1, 2:1, and 3:1). In all the experiments, the HAuCl_4_ aqueous solution was added gradually, at a rate of 1 mL every 30 min. The sample with the best characteristics was chosen for the subsequent characterization and the degradation of the organic dyes.

### 2.4. Characterization of AuNPs

The synthesized nanoparticles were characterized using various techniques. The synthesis and determination of the best parameters were evaluated by UV-Vis spectroscopy using a METASH 5000M spectrometer. The samples were diluted at a ratio of 1:10, using distilled water. The analyses were carried out in a wavelength range of 200 to 800 nm and a step of 1 nm. All spectra were performed using quartz cells. The presence of gold and its crystalline nature were determined by X-ray diffraction. The analysis was carried out using a Rigaku Ultima IV diffractometer with Cu Kα radiation in a range of 20 to 80°.

The size of the gold nanoparticles was determined by dynamic light scattering (DLS) using a Litesizer 500 Anton Paar in automatic mode. A semiconductor laser diode (λ = 658 nm) with a fixed side angle (90°) and a disposable cell were used. The sample temperature was 25 °C and the measurement duration was 10 s. The colloidal stability was evaluated with a zeta potential analysis using a Litesizer 500. The analysis was performed at 25 °C using Anton Paar Univette reusable cuvettes. Aqueous solutions of 1 M HNO_3_ or 1 M NH_3_OH were used to adjust the pH value. The samples were evaluated in triplicate, and the average of the measurements was determined. The morphological characterizations of the samples were carried out using a SEM/STEM Hitachi SU8230 cold-field emission scanning electron microscope (Hitachi High-Tech, Tokyo, Japan). The samples were prepared by dispersing a solution of colloidal nanoparticles and dropping this over the carbon-coated copper grids. The organic compounds involved in the synthesis process were proposed by evaluating the functional groups determined by FTIR analysis, recorded from 400 to 4000 cm^−1^, using a Perkin Elmer Spectrum Two FTIR spectrometer. Finally, the concentration of AuNPs was determined by TGA analysis using a Mettler Toledo TGA/DSC 2+ thermal analyzer. The temperature range employed was 30–700 °C with a heating rate of 10 °C/min.

### 2.5. Evaluation of Photocatalytic Properties

The catalytic properties of the AuNPs were evaluated by means of the degradation of methylene blue, methyl orange, and methyl red. The dyes were prepared in an aqueous solution at 5 µg∙mL^−1^. The degradation was carried out by mixing AuNPs with 1 mL of the organic dye and 10 µL of NaBH_4_. The volume of the nanoparticles was varied at 10, 30, 50, 70, and 90 µL. The evolution of the degradation was monitored by UV-Vis, analyzing the intensity of the absorbance signal of the evaluated dye, and relating this to the respective concentration through a calibration curve previously constructed with a high correlation coefficient (*R*^2^ > 0.95). The degradation capacity *q* (µg∙mg^−1^) was determined using the following equation:

(1) $$q=\frac{V}{m}\left(C_{0}-C\right)$$

where *V* is the volume of the organic dye used, in mL, *m* is the mass of the AuNPs used for photocatalytic evaluation (this can be obtained from TGA data) in mg, and *C*_0_ and *C* are the dye concentrations at the initial time, and at a given time, in µg∙mL^−1^.

#### Kinetics Model for Photocatalytic Evaluation

Kinetics models can give important information regarding the adsorption pathway and probable mechanism involved for dye degradation in the photocatalytic activity of the AuNPs. Four models were used to identify the adsorption process, pseudo-first order (PFO), pseudo-second order (PSO), Elovich model, and Weber’s intraparticle diffusion (IPD). The kinetic constants of adsorption were calculated for the various models, and the linear regression correlation coefficient (*R*^2^) values were compared to evaluate the best fit model. The Lagergren pseudo-first order model (PFO) is represented by:

(2) $$\log\left(q_{e}-q_{t}\right)=\log\left(q_{e}\right)-\frac{k_{1}}{2.303}t$$

where *q_e_* and *q_t_* (µg∙mg^−1^) are the amounts adsorbed at equilibrium and at time *t*, respectively; *k_1_* is the equilibrium rate constant in the pseudo first-order model (min^−1^).

The Ho and McKay pseudo-second order model (PSO) follows the expression:

(3) $$\frac{t}{q_{t}}=\frac{1}{k_{2}q_{e}^{2}}+\frac{t}{q_{e}}\;\;\;\mathrm{with}\;\;h=k_{2}q_{e}^{2}$$

where *h* is the initial sorption rate, and *k*_2_ is the constant equilibrium rate of the pseudo second-order model (mg∙µg^−1^∙min^−1^).

The Elovich kinetic model is expressed by the equation:

(4) $$q_{t}=\frac{1}{\beta }\ln\left(\alpha \beta \right)+\frac{1}{\beta }\ln\left(t\right)$$

where *α* is the initial adsorption rate, and *β* is the desorption constant.

Finally, the intraparticle diffusion model (IPD) follows the equation:

(5) $$q_{t}=k_{i}t^{0.5}+C_{i}$$

where *k_i_* is the intraparticle diffusion rate (µg∙mg^−1^∙min^−1^) and *C_i_* is a constant (µg∙mg^−1^).

## 3. Results

AuNPs are susceptible to surface plasmon resonance. They emit a signal, also called the absorption peak or band, in the ultraviolet-visible spectrum. This signal appears between 500–600 nm, depending on the physical characteristics of the nanoparticles, such as size, shape, and concentration [20,34].

**Figure 1:**
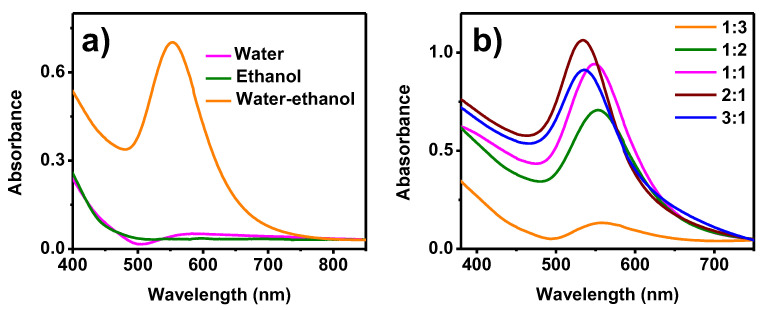
UV-Vis analysis of AuNPs synthesized with varying extracts of *Sargassum* spp., (**a**) the solvent employed for the extract preparation, and (**b**) the extract/precursor salt ratio.

Figure 1a shows the UV-Vis analysis of the different solvents used to prepare the *Sargassum* spp. extract. The ethanol extract shows no evidence of an absorption band, meaning that there were no AuNPs in this sample. In the spectrum corresponding to the synthesis using an aqueous extract, an absorption band at 585 nm was observed, suggesting the presence of AuNPs. However, the signal is very weak and broad, meaning the concentration of nanoparticles is low and the size distribution wide. Finally, the 50% water/ethanol sample had the highest concentration of small AuNPs as seen in the high-intensity absorption band on the spectrum, centered at 553 nm. As the concentration and composition of the extracts depend directly on the solvents used, the solvent that gives the greatest amount of reducing agents and stabilizers must be determined. It has been reported that only tannins are obtained in aqueous extracts, while in various alcoholic extracts phenols and glycosides are obtained [35]. Recent studies have also demonstrated that when using a combination of H_2_O and acetone (30:70% *v*/*v*), phenolic acids, monosaccharides, and glycosides are obtained from *Sargassum* spp. [36]. These findings are consistent with the present results, since it was observed that a synergic effect of using solvents with different polarities in the infusion extraction was required to obtain adequate quantities of organic compounds that can reduce Au^3+^ to Au^0^ in comparison to that using pure solvents. The 50% water/ethanol extract had the highest concentration of organic compounds with reducing and stabilizing capacity, and the use of this extract lead to the formation of a large concentration of AuNPs with homogeneous size and morphology.

The synthesis reaction can be optimized by increasing the reducing agents, in this case, the *Sargassum* spp. extract. Figure 1b shows the UV-Vis spectra obtained for various extract/salt ratios. In the first three ratios (1:3, 1:2, and 1:1), as the volume of the extract increased, higher absorption bands were observed, suggesting an increase in the concentration of AuNPs. These spectra were centered at approximately the same wavelength (553 nm), indicating a similar average size. When a 2:1 ratio of extract/salt was used, the band of the greatest intensity, centered at 534 nm, was observed. The shift towards smaller wavelengths indicates a decrease in average particle size, which is due to the increase in the reducing and stabilizing agents available in the extract [37,38]. Finally, the spectrum corresponding to a 3:1 volumetric ratio showed an absorption band at the same wavelength as the previous sample. However, its intensity was significantly less, suggesting a lower concentration of AuNPs. According to several reports, this behavior is attributed to the large number of organic compounds contained in the extracts, making the system more complex and causing poor control of the synthesis reaction. [19,39]. Therefore, increasing the amount of extract will not always lead to an improvement in nanoparticles synthesis. Based on these results, it can be deduced that the AuNPs with the best characteristics are obtained when using a 50% water/ethanol extract, with a 2:1 extract/salt ratio.

**Figure 2:**
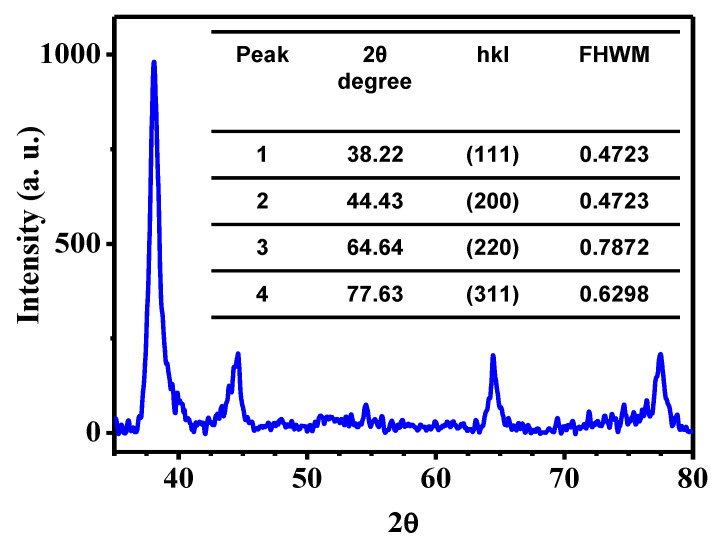
X-Ray diffraction pattern of AuNPs synthesized with water/ethanol *Sargassum* spp. extract.

Figure 2 shows the X-ray diffraction pattern corresponding to the AuNPs sample. Four different reflections can be observed, at 38.22°, 44.43°, 64.64, and 77.63°, which correspond to the crystallographic planes (110), (111), (200), and (210), respectively. This diffractogram was indexed with the card JCPDS 04-0784, corresponding to gold with a face-centered cubic structure (FCC). The crystal size of each peak was analyzed using the Williamson–Hall method, finding an average value of 14 nm. These results demonstrate the presence of gold of a crystalline nature. Finally, the weak reflections that appear are attributed to the organic compounds present in the sample, as was reported previously [13].

**Figure 3:**
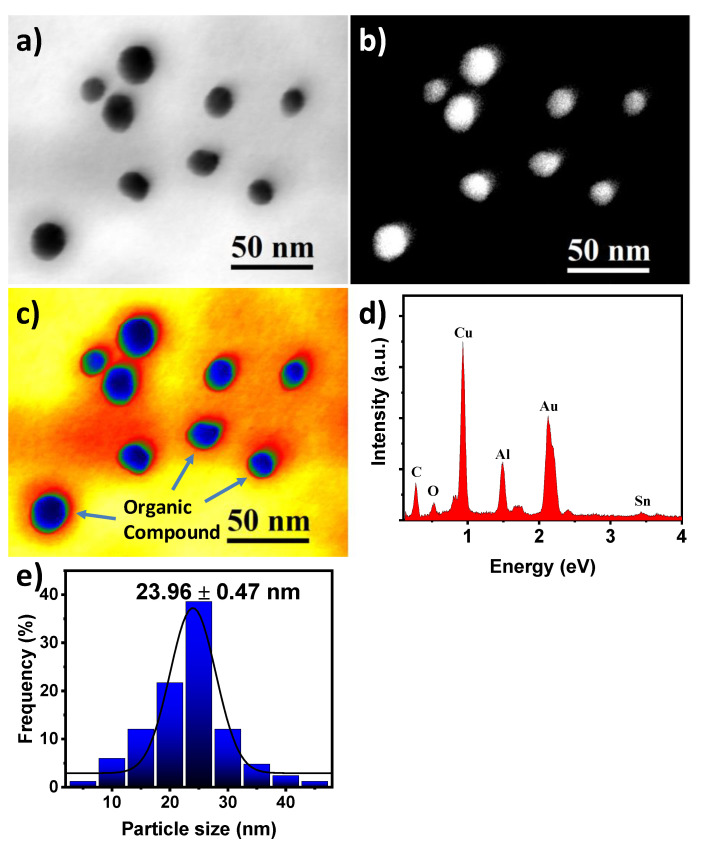
STEM micrographs of AuNPs synthesized using *Sargassum* spp., (**a**) BF-STEM image, (**b**) ADF-STEM image, (**c**) CLUT image, (**d**) EDS analysis, and (**e**) the corresponding histogram of particle size distribution.

The main challenge in producing nanoparticles is reaching a homogeneous morphology and good dispersion of them. Figure 3a shows the bright-field scanning/transmission electron microscopy (BF-STEM) image of the green synthesized AuNPs. As can be observed in the image, the AuNPs had a quasi-spherical shape with an average particle size of 23.96 ± 0.47 nm (Figure 3e). Homogeneous particle size distribution with no aggregates or agglomeration were observable, indicating success in controlling the AuNPs size via green synthesis using *Sargassum* spp. extracts. Figure 3b shows the annular dark-field (ADF)-STEM image, in which the AuNPs appear to brightly contrast because of incoherent scattering. It is important to mention that in the ADF-STEM (also called Z contrast) image, the AuNPs have a higher atomic number than that of the C from organic compounds and support, and they are shown as bright dots. To visualize the contrasting of the organic compounds from the *Sargassum* spp., the BF-STEM image was colored to highlight subtle variations in intensity, which may be difficult to discern in a grayscale image. Figure 3c shows the color look-up table (CLUT), where it can be seen clearly that the AuNPs are covered by an organic compound, which helps avoid agglomeration and aggregation. The aggregation or agglomeration of nanoparticles reduces the potential of catalytic properties due to the restriction of the interfacial area. Figure 3d shows the EDS analysis of the AuNPs in which the elemental composition of the sample is appreciated. The presence of gold confirms the composition of the nanoparticles, as the only metallic phase. The signals of the other elements (Cu, Al, and Sn) come from the sample holder used. Elements from the metallic salt and extract such as Cl, K, and As are not present in the spectrum, indicating that the cleaning process of the AuNPs is efficient and the obtained results are the product of the interaction of the nanoparticles with the dyes.

**Figure 4:**
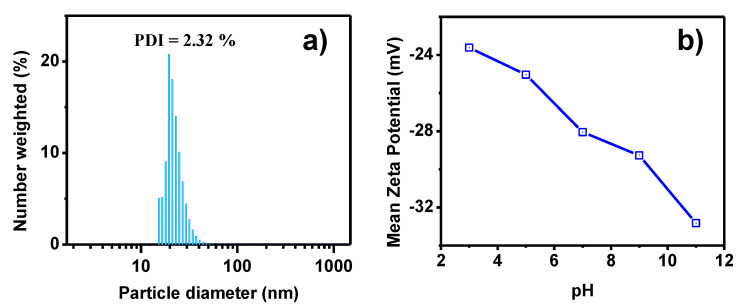
(**a**) DLS histogram and (**b**) zeta potential analysis of AuNPs synthesized with water/ethanol *Sargassum* spp. extract.

The DLS technique was used to determine the particle size and size distribution profile of the AuNPs. Figure 4a shows the DLS histogram of the AuNPs. As can be seen, the average particle size was 22.27 nm, with a standard deviation of 3.4 nm. The polydispersity index (PDI) indicates the breadth of the size distribution, where a value of 10% or less implies that the sample is monodisperse. Here, the PDI calculated was 2.32%, indicating a monodisperse size distribution. Zeta potential estimates the surface charge, which can be employed to understand the physical stability of the AuNPs in colloidal solution. Figure 4b shows the charge value against pH. High positive or negative zeta potentials suggest good physical stability, where 30 mV is considered optimum, but between 20 and 40 mV provides the system with stability and is less prone to form aggregates or increase the particle size. Therefore, the AuNPs will tend to agglomerate more easily with an acid medium than with an alkaline one. At a pH of over 10, the AuNPs are completely stable.

Concerning the catalytic activity, methylene blue is an alkaline dye, methyl orange is almost neutral, while methyl red is considered acid. As the surface area of the nanoparticles is greater in alkaline media, this suggests that the AuNPs will be more active against methylene blue and less active against methyl red.

The organic compounds from the *Sargassum* spp. extract, involved in the reduction and stabilization process, were determined using FTIR spectrometry.

**Figure 5:**
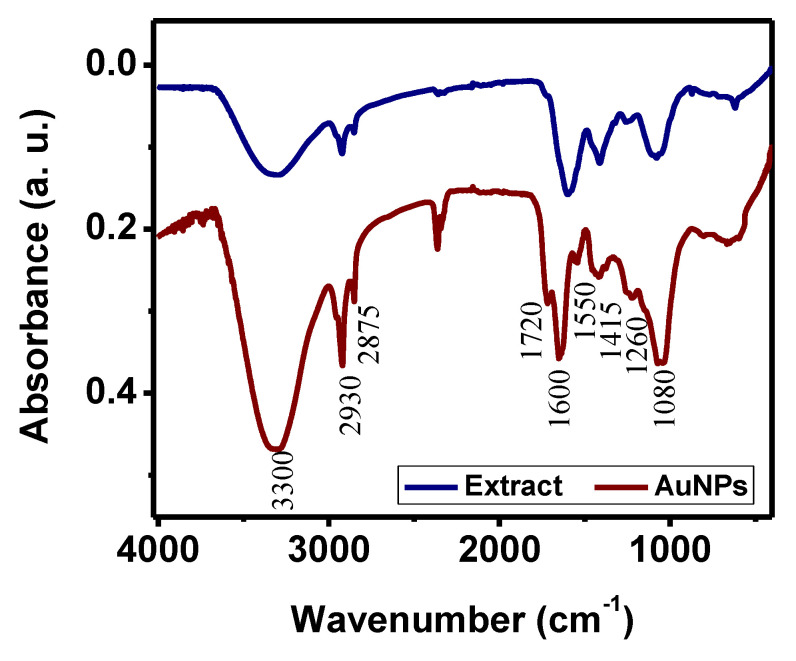
FTIR analysis of water/ethanol *Sargassum* spp. extract and AuNPs.

The AuNPs and the *Sargassum* spp. spectra are shown in Figure 5. As can be seen, both spectra are quite similar, suggesting that the surface of AuNPs is covered by functional groups of *Sargassum* spp. extract. Nevertheless, the AuNPs spectrum shows a slight shift in some absorbance bands, which suggests that those functional groups were involved in the synthesis [40]. Looking at the results in detail, it is seen that both samples were composed of hydroxyl compounds, which caused the characteristic broad absorption band between 3650 and 3250 cm^−1^. The absorption bands at 2935 and 2860 cm^−1^ can be attributed to long-chain linear aliphatic compounds. Then, at lower wavenumbers, a small band centered at 1720 cm^−1^ was caused by simple carbonyl compounds.

Macroalgae, such as *Sargassum* spp., have a high content of aromatic polyphenol complex or lignin-like materials [28]. In the FTIR spectrum corresponding to *Sargassum* spp. extract, the absorption bands caused by aromatic rings are observed at 3150–3000 cm^−1^ [41]. However, this vibrational signal overlaps with the OH absorption band. Hence, the presence of this kind of compound can be confirmed by the bands centered at 1600 and 1550 cm^−1^, corresponding to aromatic ring vibrations [41]. The sample also included olefinic groups established by the absorption band between 1420–1410 cm^−1^ assigned to vinyl C-H in-plane bend vibrations. The absorption band between 1270–1230 cm^−1^ was due to the presence of aromatic ethers on the *Sargassum* spp. extract. Finally, the band at 1080 cm^−1^ was caused by the C-O stretch of primary alcohol. Accordingly, it is possible to conclude that the polyphenolic groups are the main reducing agents in the biosynthesis of AuNPs. Other functional groups from biomolecules presented in the *Sargassum* spp. extract are also confirmed for the stabilization of AuNPs.

Determining the concentration of AuNPs present in the sample is very important to evaluate any application. These data can be obtained by TGA analysis, which is a progressive heating to high temperatures [42,43,44]. In this way, moisture is removed, and organic compounds are calcined. Therefore, the mass corresponding to the AuNPs can be estimated.

**Figure 6:**
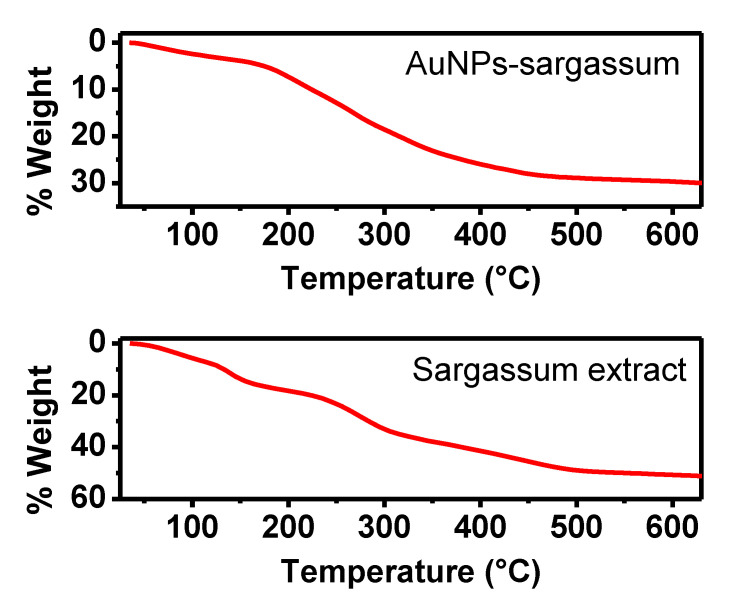
TGA analysis of AuNPs synthesized with water/ethanol *Sargassum* spp. extract.

Figure 6 shows the TGA results of the *Sargassum* spp. extract and AuNPs dry samples. A significant decrease in the percent weight of the sample was seen. At least two different events can be observed; the first, between 120 and 200 °C, attributed to the evaporation of moisture. The second event occurred between 270 and 330°, corresponding to the decomposition of other organic compounds present in the extract. The calcination then continued with a linear behavior until reaching 500 °C. According to Elbagory et al., the weight loss in this temperature range is attributed to the decomposition of resistant aromatic compounds [45]. After 500 °C, there were no obvious changes in weight, indicating a complete calcination of the extract. Similar thermal behavior has been reported in the synthesis of nanoparticles by green attempts [44,46]. This analysis showed a weight loss of 51%, meaning that the remaining 49% corresponds to the weight of carbon and calcined products.

On the other hand, the TGA analysis of AuNPs was carried out using 5.03 mg obtained by drying 10 mL of sample. A decrease in weight from 30 to 500 °C was observed, attributed to the decomposition and calcination of the organic compounds present in the sample. After this temperature, there were no significant changes in the weight of the sample, whose loss was equivalent to 29%, corresponding to 1.46 mg. Therefore, the remaining weight was equal to 3.57 mg, of which 1.75 mg (49%) corresponded to the organically calcined products. That is, 1.82 mg of the sample corresponded to the uncalcined phase, which in this case was AuNPs since, according to X-ray and EDS analysis, the only metallic phase present in the sample was gold. Therefore, taking into account the volume of sample used, it can be determined that the concentration of nanoparticles was equal to 0.182 mg/mL.

**Figure 7:**
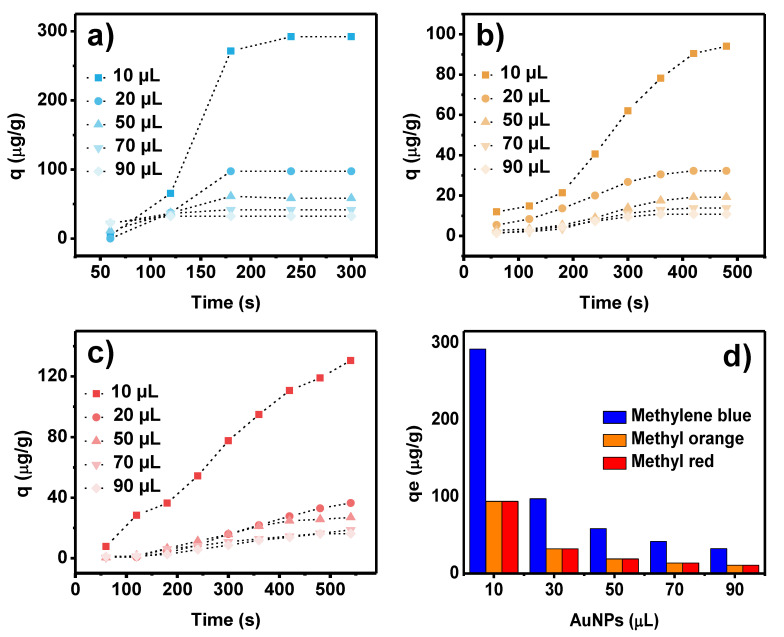
Time-dependent degradation capacity for (**a**) methylene blue, (**b**) methyl orange, and (**c**) methyl red at 25 °C; (**d**) equilibrium degradation capacity *q_e_* (µg∙mg^−1^) for different volumes of AuNPs used for photocatalysis.

The results of the organic dye degradation expressed as the increased function of adsorption capacity over time are seen in Figure 7a–c. It can be observed that the degradation of the dyes was extremely fast, less than ten minutes, with methylene blue being the fastest to degrade (~5 min) and methyl red the slowest (~9 min).

Figure 7d shows the effect of varying the initial volume of AuNPs used for dye degradation. As the volume increases, the equilibrium adsorption capacity (*q_e_*) is reduced, indicating, the optimal concentration of AuNPs (for this case, 2.75 µg∙mL^−1^) required for the catalytic process. Consequently, beyond this, there will be an excess of nanoparticles participating in the catalytic process, accelerating the reaction but at the expense of underusing AuNPs.

It is also observed in Figure 7 that the AuNPs have a higher preference for methylene blue degradation, compared to that for methyl orange and methyl red, confirming the suggestion proposed from the zeta potential, that AuNPs will have higher catalytic activity for cationic dyes since they have the highest surface area in alkaline media.

The characteristic parameters of each proposed model were obtained after the linear plot of the equation described in the Experimental section, and their values with their correlation coefficient are shown in Table 1.

As observed from the correlation coefficient of the kinetics models, the best fit is from de Weber’s intraparticle diffusion model (IDP), which is usually the third selection after PFO and PSO for liquid degradation kinetics in environmental remediation. It is noticeable that methylene blue degradation is controlled only by intraparticle diffusion since the linear fit from the model passes through the origin (*C_i_* = 0). Usually, the *C_i_* value is related to information about the thickness of the boundary layer. The larger C implies the more significant effect of the boundary layer. This is important when negative intercepts are obtained since boundary layer thickness related to surface reaction control is retarding IDP.

**Figure 8:**
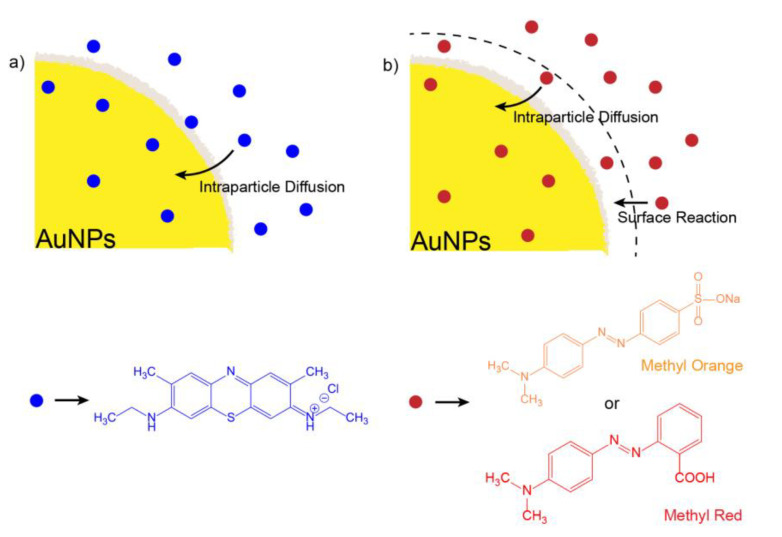
Scheme of the degradation mechanism proposed for (**a**) methylene blue and (**b**) methyl orange/red organic dye.

For methyl orange and methyl red, the initial degradation rate (at very short times) is governed by a surface reaction and then by IDP. A scheme for the photocatalytic dye degradation process is presented in Figure 8. Taking into account the use of NaBH_4_, the complete degradation mechanism can be explained as follows. First, BH_4_^−^ ions are adsorbed on the AuNPs’ surface. Subsequently, the AuNPs reduce the kinetic barrier by lowering the reaction activation energy while the dye molecules diffuse into their surface. Thus, reductive degradation becomes thermodynamically and kinetically favorable. When the kinetic barrier is overcome, the AuNPs act as a reservoir for the electrons, allowing the excess electrons from the surface of the nanoparticles to transfer to the dye molecules and reduce them [47,48].

Table 2 shows the turnover frequency (TOF) for the different dyes with the lowest and highest concentrations of AuNPs used to degrade each dye. It is observed that TOF has the same tendency as that of the adsorption capacity (*q* [µg∙mg^−1^]); as the AuNPs concentration increases, the value of TOF is reduced. Again, these TOF values for dye degradation are consistent with values reported elsewhere for other nanoparticles [49,50].

On the other hand, the percentage of degradation was obtained using 90 μL of AuNPs. Efficiencies of 99.6, 98.2, and 94.9% were obtained to degrade methylene blue, methyl red, and methyl orange, respectively. Table 3 shows a comparison of the degradation of organic dyes using AuNPs synthesized by different routes. As can be seen, the results obtained in this work show high efficiencies, comparable or even better than other reported results. In addition, it should be noted that the synthesis of nanoparticles by green routes has certain advantages over other synthesis techniques since they are simple, inexpensive, and non-toxic processes. Comparing the results obtained with biosynthesized nanoparticles, the use of Sargassum should be highlighted. The overpopulation of this alga has brought severe environmental and economic problems, so this practical and sustainable use for the synthesis of AuNPs could mean a decrease in the nuisance caused by Sargassum in the Mexican Caribbean.

**Figure 9:**
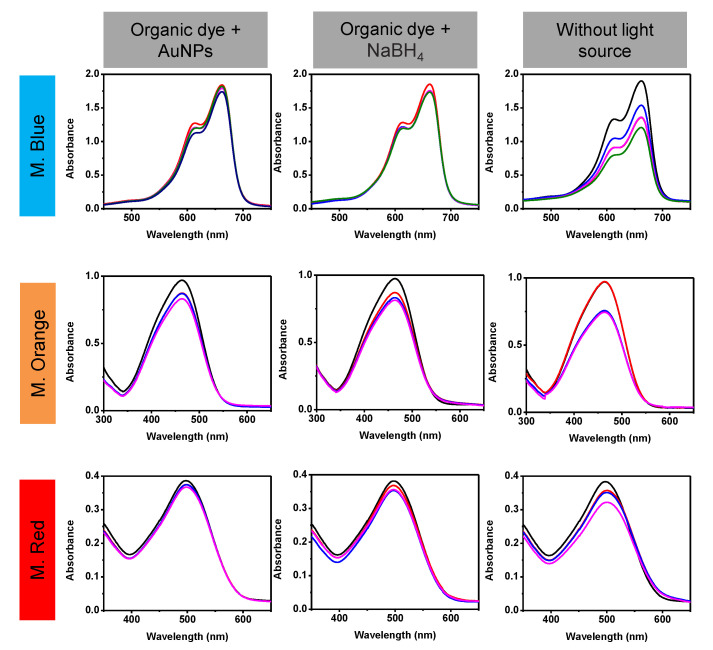
UV-Vis analysis of the blind tests for methylene blue, methyl orange, and methyl red degradation.

To demonstrate that the catalytic activity is a combination of the properties of the AuNPs, NaBH_4_, and the source of visible, ultraviolet light, UV-Vis analysis of the different blind tests was carried out, see Figure 9. The following combinations were evaluated: (a) AuNPs with dyes, (b) NaBH_4_ with dyes, and (c) AuNPs with NaBH_4_ and dyes but without a light source. UV-Vis spectra were taken every 3 min and carried out in the dark. The behavior of the three dyes was similar when mixed with a single reagent (NaBH_4_ or AuNPs); a very small decrease in the absorption band of the dye was observed, meaning little degradation. A decrease in the absorption band was observed when the degradation was carried out in the absence of the UV-Vis source, corresponding to degradations between 10 and 20%, after 9 min. These results demonstrate the ability of AuNPs with NaBH_4_ to degrade organic dyes, and that the reaction rate can be increased by using a UV-Vis light source.

**Figure 10:**
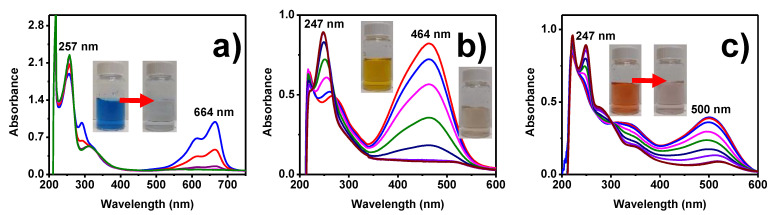
UV-Vis analysis of the degradation of (**a**) methylene blue, (**b**) methyl orange, and (**c**) methyl red using AuNPs.

The organic dyes once degraded are colorless and non-toxic. The reaction products emit absorption peaks in the UV-Vis spectrum in the range 200–300 nm. The Figure 10 shows the UV-Vis spectra in a wide wavelength range (200–700 nm), corresponding to the degradation of the dyes using the highest volume of AuNPs (90 μL). Figure 10a corresponds to the degradation of methylene blue. The characteristic signal of this dye was at 664 nm, which decreased as time passed, signifying its gradual degradation. At the same time, a signal at 257 nm increased in intensity. Some reports link this signal to the product formed, that is, leuko methylene blue [60,61].

Similar behavior was observed for the degradation of methyl orange and methyl red (Figure 10b,c). The absorption signals of methyl orange (464 nm) and methyl red (500 nm) decreased as degradation occurred. However, a new signal, at 247 nm, began to increase in intensity. This signal corresponds to organic compounds with amino groups (-NH_2_). According to Sha et al., this band appears as a result of the degradation of the azo bond of this type of organic dye [62].

**Figure 11:**
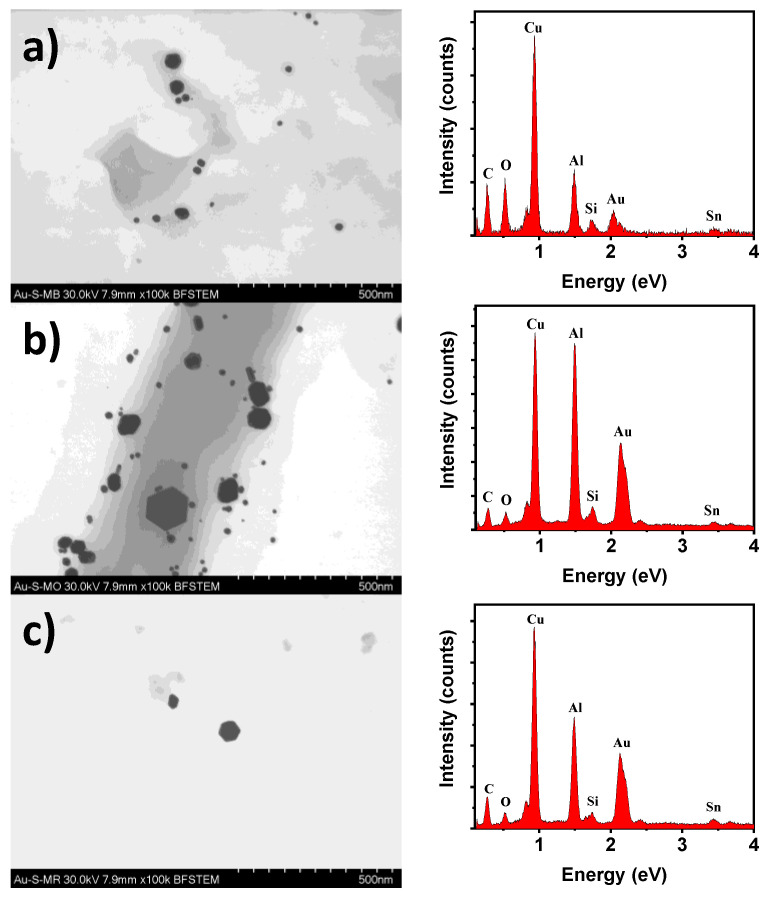
SEM and EDS analysis of the AuNPs after degradation of (**a**) methylene blue, (**b**) methyl orange, and (**c**) methyl red.

Figure 11a–c show the scanning electron microscopy (SEM) images with their respective EDS spectrum of the AuNPs after the degradation of methylene blue, methyl orange, and methyl red, respectively. As can be observed from the SEM images, agglomerates and aggregates of AuNPs are not present, indicating a good particle size distribution. With respect to the particle size, apparently the AuNPs particle size was preserved in the samples after the degradation of methylene blue and methyl red. However, in the sample after the degradation of methyl orange, two particle size distributions were observables, the first one, AuNPs with a particle size similar to that observable in the samples after the degradation of methylene blue and methyl red, and the second one, faceted AuNPs of a larger size as a product of growth kinetics. On the other hand, the EDS spectra only show Au elements from the nanoparticles, C and O are from the organic compound and the support film of the grid, Cu, Al, Si, and Sn elements are from the holder and grid.

## 4. Conclusions

From the results of this work, it is seen that *Sargassum* spp. contains compounds with antioxidant capacity that can reduce gold ions and subsequently stabilize nanosized particles. The catalytic efficiency of metal nanoparticles increases as their surface-to-volume ratio increases. Therefore, various parameters such as the solvent used to make the *Sargassum* extract and the extract/precursor salt volume ratio were evaluated to optimize the synthesis condition to obtain high yield, stable, small, and monodisperse nanoparticles. The results showed that the highest concentration of antioxidant compounds is extracted using a 50% water/ethanol mixture, leading to the synthesis of a higher concentration of homogeneous AuNPs. The increase in the extract volume leads to a more efficient synthesis, obtaining the best results from a 2:1 ratio of extract/precursor salt. In this condition, nanoparticles are spherical and range in size from 15 to 30 nm, with results corroborated by DLS and SEM. According to the FTIR results, the reduction and stabilization capacity is attributed to the polyphenolic compounds from *Sargassum* spp.

Finally, the AuNPs were used for the reduction of toxic organic dyes. The results were excellent in efficiency and degradation for methylene blue, methyl orange, and methyl red. The kinetic study shows that the degradation of methylene blue is due to intra-particle diffusion. In contrast, methyl orange and methyl red degradations were carried out in two stages: first, by a superficial reaction, and second, by intraparticle diffusion. This finding is important because it shows that the stabilizing agent does not compromise the catalytic efficiency of AuNPs, making the use of *Sargassum* spp. promising for the synthesis of gold nanoparticles. In this way, we can make use of the overpopulation of these algae in the Mexican Caribbean and offer an excellent alternative for the degradation of toxic substances, which can be applied in water treatment.

## Figures and Tables

**Table 1:** Characteristic parameters obtained for the degradation of the organic dyes with the different kinetic models.

	Methylene Blue			Methyl Orange			Methyl Red		
Kinetic Model	Characteristic Parameter		*R* ^2^	Characteristic Parameter		*R* ^2^	Characteristic Parameter		*R* ^2^
PFO	*k_1_*	1.3150	0.6339	*k_1_*	0.4776	0.7787	*k_1_*	0.3344	0.9198
	*q_e_*	1533.10		*q_e_*	226.05		*q_e_*	224.70	
PSO	*k_2_*	0.0094	0.3937	*k_2_*	0.0003	0.1862	*k_2_*	0.0002	0.3453
	*h*	5.5036		*h*	8.1037		*h*	9.9900	
	*q_e_*	−24.1546		*q_e_*	−172.4138		*q_e_*	−200	
Elovich model	*α*	194.1453	0.8460	*α*	37.7868	0.8148	*α*	49.4101	0.9098
	*β*	0.0049		*β*	0.0226		*β*	0.0171	
IPD	*k_i_*	1	1	*k_i_*	52.0833	0.9149	*k_i_*	64.6271	0.9768
	*C_i_*	0		*C_i_*	−54.4762		*C_i_*	−65.3418	

**Table 2:** Turnover frequency (TOF) for the dyes showing the lowest and highest concentrations of AuNPs.

Dye	AuNPs (µL)	TOF (h^−1^)
Methylene Blue	10	3.60
	90	1.07
Methyl Orange	10	4.98 × 10^−1^
	90	6.18 × 10^−2^
Methyl Red	10	7.75 × 10^−1^
	90	0.96 × 10^−1^

**Table 3:** Comparison of the degradation of organic dyes using AuNPs synthesized by different routes.

Material	Synthesis Method	Organic Dye	% Degradation	Reference
AuNPs (This work)	Green synthesis	Methylene blue	99.6	
		Methyl orange	98.2	
		Methyl red	94.9	
AuNPs	Chemical reduction	Methylene blue	99.4	[51]
		Methyl orange	91	
AuNPs-zeolite	Chemical reduction	Methylene blue	50	[52]
AuNPs-MoS_2_	Chemical reduction	Methylene blue	94.3	[53]
		Methyl red	96.7	
AuNPs	Green synthesis	Methylene blue	96.26	[37]
Au-Ag alloy NPs	Green synthesis	Methylene blue	96.59	[54]
		Methyl orange	80.06	
		Methylene violet	86.88	
AuNPs	Green synthesis	Methylene blue	84	[55]
Au-ZnO NPs	Sol-gel	Methylene blue	82.1	[56]
AuNPs	Green synthesis	Acid Orange II	94.6	[38]
		Acid yellow II	92.7	
		Reactive red	95.3	
AuNPs	Green synthesis	Methyl orange	83.25	[57]
		Rhodamine B	87.64	
AuNPs	Green synthesis	Methylene violet	89.17	[58]
TiO_2_-AuNPs	Chemical reduction/UV irradiation	Methylene blue	90	[59]

## Data Availability

The data presented are available in this study: Green synthesis of homogeneous gold nanoparticles using *SargassumSargassum* spp. extracts and their enhanced catalytic activity for organic dyes.
